# Clinical Outcomes after Bilateral Implantation of Trifocal Diffractive Intraocular Lenses and Extended Depth of Focus Intraocular Lenses

**DOI:** 10.3390/jcm11195729

**Published:** 2022-09-27

**Authors:** Kyoung Yoon Shin, Dong Hui Lim, Tae-Young Chung

**Affiliations:** 1 Nune Eye Hospital, Seoul 06198, Korea; 2 Department of Ophthalmology, Sungkyunkwan University School of Medicine, Samsung Medical Center, Seoul 06351, Korea; 3 Department of Clinical Research Design & Evaluation, Samsung Advanced Institute for Health Sciences and Technology, Sungkyunkwan University, Seoul 06351, Korea

**Keywords:** multifocal, trifocal, extended depth of focus, extended range of vision

## Abstract

The purpose of this retrospective study is to investigate clinical outcomes of bilateral implantation of diffractive trifocal intraocular lenses (IOLs) and extended depth of focus IOLs in Koreans. The clinical outcomes of cataract surgery with bilateral implantation of PanOptix, FineVision, Symfony, and MiniWell were evaluated. Uncorrected distant, intermediate (80 cm, 60 cm), near (40 cm) visual acuity, defocus curve, manifest refraction, contrast sensitivity, and higher-order aberrations, quality of vision, spectacle independence, and subjective satisfaction at postoperative 3 months were assessed. A total of 136 eyes in 68 patients were included in the analyses. PanOptix and FineVision performed better visual acuity compared to Symfony and MiniWell at 40 cm distance. Defocus curve showed broad range of vision in PanOptix and FineVision with visual acuity of more than 0.1 logarithm of the minimum angle of resolution at −2.5 diopter (D) of defocus power, while Symfony and MiniWell presented excellent intermediate vision without a dip at defocus power of −0.5 D to −1.0 D. Glare, halo, and starburst were significantly less in MiniWell compared to others. In conclusion, all four IOLs presented satisfactory clinical outcomes. PanOptix and FineVision provided good near and intermediate vision, while Symfony and MiniWell provided good intermediate vision. MiniWell induced little dysphotopsia.

## 1. Introduction

In recent times, the objective of cataract surgery has shifted from simply gaining precise refractive outcomes to also correcting presbyopia. Multifocal intraocular lenses (IOLs) have gained popularity for this purpose, and various multifocal IOLs are being manufactured by different companies, providing a broad range of options in IOLs [1,2].

Classic multifocal IOLs are diffractive bifocals and, thus, provide two working distances (distant and near) [3]. Diffractive bifocal IOLs with additional power are selected according to the patients’ lifestyles. Although diffractive bifocal IOL implantation is an effective way to satisfy patients who want to stop using their glasses after cataract surgery, it often results in suboptimal intermediate visual acuity, which is more important than near and distant visual acuities [4,5] for working on the computer or simple household chores.

Diffractive trifocal IOLs and extended depth of focus (EDOF) IOLs have been recently introduced to solve the aforementioned problem. Diffractive trifocal IOLs have three foci and, hence, provide a wider range of spectacle independence, especially at an intermediate distance, than bifocal IOLs [6,7]. EDOF IOLs have also been recently developed and are a promising technology for obtaining good visual outcomes and spectacle independence in patients at different distances while minimizing visual disturbances, which are commonly associated with multifocality [8].

In the current study, the investigators compared the clinical outcomes of bilateral implantation with diffractive trifocal IOLs and EDOF IOLs. These procedures were performed at a single clinical center, and we assessed the advantages and disadvantages of each IOL.

## 2. Methods

### 2.1. Study Design

This is a retrospective, comparative, and interventional study. The medical records of all consecutive patients who underwent cataract extraction and bilateral implantation of trifocal IOLs or EDOF IOLs between January 2016 and December 2018 at the Samsung Medical Center were retrospectively reviewed. The IOLs included in the study were PanOptix (Alcon, Fort Worth, TX, USA), FineVision POD F (PhysIOL, Liege, Belgium), Symfony (Abbott Medical Optics, Santa Ana, CA, USA), and MiniWell (SIFI, Catania, Italy). Patient information was extracted from the medical records, including age, sex, baseline optical biometry, implanted IOLs, and postoperative visual outcomes and complications. Any patients with a previous history of ocular trauma, surgery, and other ocular diseases that might interfere with the outcomes of cataract surgery were excluded from the analysis. This study was performed in accordance with the Declaration of Helsinki and was approved by the Samsung Medical Center Institutional Review Board (permission number: SMC 2019-07-162). The board waived the requirement for informed consent based on the retrospective design of the study.

### 2.2. Intraocular Lenses

The PanOptix IOL and the POD FineVision IOL are both categorized into aspheric diffractive trifocal IOL. Several differences exist between the two products, but the most practical difference is the target distance of focus. The PanOptix IOL have distant, intermediate (60 cm), and near (40 cm) foci. The POD FineVision IOL, on the other hand, have additional powers of +1.75 D and +3.50 D in the IOL plane which correspond to 40 cm and 80 cm in distance [9]. The POD FineVision also provides a trifocal lens with toricity. POD FineVision IOLs without a toric component are manufactured under the FineVision POD F name, whereas the toric versions of POD FineVision IOLs are called FineVision POD FT.

The Symfony IOL is a diffractive IOL with EDOF performance and the MiniWell is a refractive lens by asphericity modulation with EDOF performance. The Symphony IOL uses diffractive echelette optic design to elongate the depth of focus and compensates for the corneal chromatic aberration. Meanwhile, the MiniWell IOL uses three different optical zones with different spherical aberrations to generate a continuum of foci and increase the depth of focus.

### 2.3. Preoperative Assessment, Surgery, and Postoperative Evaluation

The routine process for bilateral implantation of multifocal IOLs followed at the Samsung Medical Center was previously described in other published papers [10,11]. The brief explanation of the schedule and the process is listed below.

Preoperatively, the detailed clinical histories were made in the outpatient clinic of the two surgeons (D.H.L. and T-Y.C.) and all patients underwent full ophthalmologic examinations, including uncorrected distance visual acuity (UDVA), uncorrected intermediate visual acuity (UIVA) at 60 cm and 80 cm, uncorrected near visual acuity (UNVA) at 40 cm, and corrected distance visual acuity (CDVA) under photopic conditions (85 candelas (cd)/m^2^), as well as slit-lamp microscopy, intraocular pressure, and fundus examinations. Anterior chamber depth, axial length, and keratometric values were evaluated using the IOL Master (version 5.4, Carl Zeiss Meditec, Dublin, CA, USA). Emmetropia was the target refraction condition, except for Symfony. For the Symfony group, the target refraction for dominant eye was ‘emmetropia’ and for the non-dominant eye was −0.5 to −0.75 D to enhance the near visual acuity. To select the appropriate lens diopter (D), the SRK/T, Haigis, and Barrett II formulas were all considered on the basis of individual characteristics of ocular biometry [10].

All surgeries were performed by two experienced surgeons (D.H.L. and T.-Y.C.). Standard suture-less phacoemulsification with a 2.7 mm clear corneal incision was performed under topical anesthesia. The surgeon carefully determined the incision axis based on preoperative corneal astigmatism, which was measured using the Scheimpflug topography (Oculus, Wetzlar, Germany).

Temporal corneal incisions were made in the eyes with corneal astigmatism of less than 0.5 D, and steep axis corneal incisions were performed in the eyes with corneal astigmatism equal to or greater than 0.5 D. For implantation of FineVision IOL, toric versions of the POD FineVision IOLs (FineVision POD FT) were used in patients with corneal astigmatism equal to or greater than 1.0 D. Temporal corneal incisions were made in these patients. For those with corneal astigmatism equal to or greater than 1.0 D and who planned to implant IOL other than FineVision, femtosecond laser-assisted astigmatic keratotomy was performed [12]. IOLs were implanted in the bag with a single-use injector and cartridge. In the case of toric IOL, the IOL was rotated to the targeted position by aligning the toric reference marks on the IOL surface with limbal axis marks before finishing the surgery [10].

The patients were routinely evaluated at one day, one week, one month, and three months postoperatively. During these routine ophthalmologic examinations, UCVA, intraocular pressure, and results of the slit lamp examination were recorded. At the one-month and three-month postoperative visits, the patients went through examinations for assessing visual acuities, manifest refraction, defocus curves, contrast sensitivity, higher-order aberrations, and subjective satisfaction. UDVA, UIVA at 60 cm and 80 cm, UNVA at 40 cm, and CDVA were measured. The defocus curves of distant-corrected vision were evaluated under photopic conditions using lenses with powers ranging from +2.0 D to −4.0 D, with 0.5 D increments. Visual acuities and defocus curves were recorded in both monocular and binocular conditions. Contrast sensitivity at 3, 6, 12, and 18 cycles per degree were measured using a CSV-1000 chart (Vector Vision, Greenville, OH, USA) under photopic (85 cd/m^2^) and mesopic (~3 cd/m^2^) conditions with a neutral density filter at three months after surgery. Results were converted to log units for statistical analysis using a specific table for the CSV-1000 [11,13]. An OPD-scan III aberrometer (Nidek Inc., Tokyo, Japan) was performed after pupil dilation and measured total higher-order aberrations (HoA), spherical aberration (SA), coma aberration, and trefoil aberration with a pupil of 4.0 mm diameter. All patients were asked to complete the questionnaire regarding overall satisfaction, presence of visual artifacts, and dependency on spectacles for near, intermediate, and far vision. Spectacle independency was evaluated in 10 levels: “wear glasses every time” was scored to be 10 and “never use” was scored to be 0. “Near” was defined as distance for books or smartphone, “intermediate” was defined to be computer work or house work distance, and “far” was defined to be distance for driving or outdoor activity. Overall satisfaction was evaluated using 5 levels: very satisfied (5), satisfied (4), neither satisfied nor dissatisfied (3), unsatisfied (2), very unsatisfied (1). Severity of visual artifacts was divided into four levels: none (0), minimal (1), moderate (2), and severe (3), and assessed using the modified version of the Quality of Vision questionnaire [11,14]. Patients were provided with a brochure having artifact images while participating in the questionnaire.

### 2.4. Statistical Analysis

The Statistical Package for the Social Sciences (SPSS) for Windows (version 23.0, SPSS, Inc., Chicago, IL, USA) was used for statistical analysis of the data. The clinical data at postoperative 3 months were compared among the four IOL groups. To assess the difference among outcomes, the Fisher’s exact test was used for categorical parameters and Kruskal–Wallis test was used for continuous variables. For direct comparison of PanOptix and FineVision (difference between two trifocal IOLs), and Symfony and MiniWell (difference between two EDOF IOLs), the Mann–Whitney test and Fisher’s exact test were employed. Wilcoxon signed rank test was applied to assess the significance of differences between preoperative and postoperative data. A *p*-value of less than 0.05 was considered to be statistically significant.

## 3. Results

### Baseline Characteristics

We studied 136 eyes in 68 patients (28 men (41.2%) and 40 women (58.8%)) with an average age of 60.2 ± 9.3 years (range: 40 to 78 years). All subjects underwent cataract surgery without any unwanted events or postoperative complications; 21 eyes out of 38 eyes used Toric versions of the POD FineVision IOLs to correct corneal astigmatism. Table 1 lists the baseline characteristics and biometry of each group. Patients in the FineVision group were more myopic and had longer axial length and lower UCVA preoperatively than those in all other groups. There was no significant difference in the other baseline demographics and biometry among the study groups. Of 68 individuals, 6 patients were less than 50 years of age (range, 21 to 48 years). The average best-corrected visual acuity at the time of surgery was 0.37 logMAR in these patients. One patients (21 years old) had atopic dermatitis, but the other five patients had no underlying ocular and systemic diseases related to cataracts.

**Figure 1:**
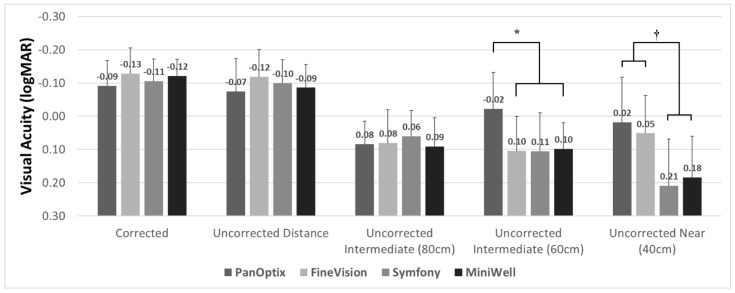
**Binocular visual acuities with PanOptix, FineVision, Symfony, and MiniWell.** PanOptix provided better uncorrected visual acuity at 60 cm distance than other intraocular lenses (*). Trifocal intraocular lenses (PanOptix and FineVision) provided better visual acuity at 40 cm than extended depth of focus intraocular lenses (Symfony and MiniWell) (†). Corrected distant, uncorrected distant, and uncorrected intermediate (80 cm) visual acuities were comparable among the four intraocular lenses. Error bar indicates standard deviation.

Figure 1 demonstrates the three-month postoperative visual acuity of the four IOL groups. All groups showed satisfactory corrected and uncorrected distant visual acuity. The corrected distant visual acuity was −0.09 ± 0.09 in the PanOptix group, −0.13 ± 0.08 in the FineVision group, −0.10 ± 0.07 in the Symfony group, and −0.12 ± 0.06 in the MiniWell group, using the logarithm of the minimum angle of resolution (logMAR) scale. Intermediate vision at 80 cm was comparable in all four groups. The PanOptix group performed better than the FineVision, Symfony, and MiniWell groups at 60 cm. Trifocal IOLs (PanOptix and FineVision) provided better visual acuity at 40 cm than the extended depth of focus IOLs (Symfony and MiniWell).

**Figure 2:**
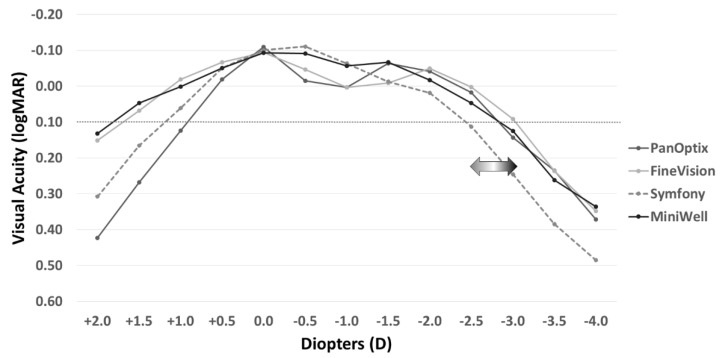
**Distant-corrected binocular defocus curves with PanOptix, FineVision, Symfony, and MiniWell.** PanOptix, FineVision, and MiniWell maintained visual acuity above 0.10 logMAR at the defocus power of −2.5 D, whereas Symfony presented with narrower depth of focus than the other IOLs. Symfony and MiniWell provided better visual acuity at the defocus power of −0.5 D and −1.0 D, and MiniWell presented with more extended defocus curve than Symfony.

Figure 2 shows the defocus curves of the four study groups. The Symfony and MiniWell groups (EDOF IOLs) presented with visual acuity greater than 0.00 logMAR at −0.5 D and −1.0 D defocus power (equivalent of near viewing at 1 m to 2 m). The curves also facilitated the maintenance of visual acuity greater than 0.1 logMAR at −3.0 D defocus power in the FineVision group, at −2.5 D defocus power in the PanOptix and MiniWell groups, and at −2.0 D defocus power in the Symfony group. The detailed data on the defocus curves including mean and standard deviation are provided in Supplemental Table S1.

The refractive outcomes of the four groups are summarized in Table 2; it also shows the final postoperative absolute prediction error, which is the absolute difference between the postoperative spherical equivalent measured by manifest refraction and the target refraction of surgery based on the SRK/T, Haigis, and Barrett II formulas. The Symfony group had more myopic outcomes than the other study groups, and the absolute prediction error did not differ among the four groups.

**Figure 3:**
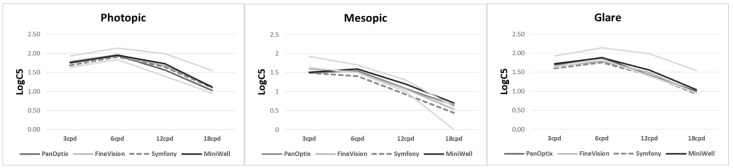
**Contrast sensitivity with PanOptix, FineVision, Symfony, and MiniWell.** There were no statistical differences tested by analysis of variance among the contrast sensitivities at any spatial frequencies (3, 6, 12, and 18 cycle per degree (cpd)) under photopic condition (*p* = 0.215, 0.847, 0.289, 0.538, respectively), mesopic condition without glare (*p* = 0.391, 0.641, 0.223, 0.254, respectively), and mesopic condition with glare (*p* = 0.365, 0.213, 0.437, 0.709, respectively).

Figure 3 shows contrast sensitivity under both photopic and mesopic conditions. There was no statistically significant difference among the groups. The detailed data on contrast sensitivity including mean and standard deviation are provided in Supplemental Table S2.

The postoperative ocular HoA are summarized in Table 3. Total HoA did not show any significant difference among the four IOL groups. Regarding SAs, the PanOptix and FineVision groups (trifocal IOLs) presented overall positive values of SA, whereas the Symfony and MiniWell groups showed negative values of spherical aberration.

**Figure 4:**
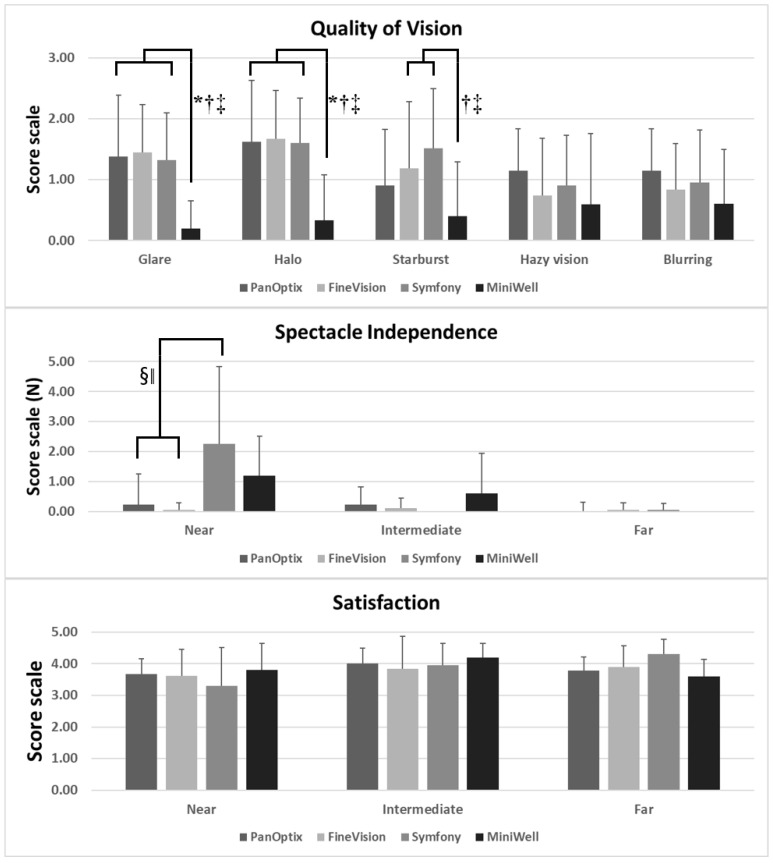
Subjective symptoms experienced with PanOptix, FineVision, Symfony, and MiniWell, as reported in the questionnaire (quality of vision, spectacle independency, and overall satisfaction). (**Top**) MiniWell presented with less glare, halo, and starburst compared with the other three intraocular lenses. (*p* = 0.004 for glare, *p* = 0.002 for halo, *p* = 0.027 for starburst by Kruskal–Wallis test). (**Middle**) Symfony presented with the greatest spectacle dependency at near distance, followed by MiniWell (*p* = 0.001 by Kruskal–Wallis test). (**Bottom**) All four intraocular lenses in the study presented with comparable overall satisfaction for near, intermediate, and far vision. Spectacle dependence score scale: 0–10 (0 = none; N = N out of 10; 10 = Always). Quality of vision score scale: 0–3 (0 = none; 1 = mild; 2 = moderate; 3 = severe). Subjective satisfaction score scale: 1–5 (1 = very unsatisfied; 2 = unsatisfied; 3 = neither satisfied nor dissatisfied; 4 = satisfied; 5 = very satisfied). Error bar indicates standard deviation. Footnotes indicate statistically significant differences among the four groups: (*: PanOptix vs. MiniWell, †: FineVision vs. MiniWell, ‡: Symfony vs. MiniWell, §: PanOptix vs. Symfony, ‖: FineVision vs. Symfony.

Figure 4 presents the spectacle independency, quality of vision, and overall satisfaction measured by questionnaires. PanOptix and FineVision provided better spectacle independency at near distance compared with Symfony and MiniWell. The MiniWell provided less glare, halo, and starburst compared with the other IOLs. All four groups showed good overall satisfaction.

Post hoc power analyses results are described in Supplemental Table S3.

## 4. Discussion

Several previous studies have reported that trifocal IOLs perform better at near distance than Symfony [15,16]. Mencucci et al., reported that PanOptix and AT LISA tri 839 MP (Carl Zeiss Meditec) provide better near visual acuity than Symfony [15]. Cochener, et al., also reported that near vision was statistically better for PanOptix and FineVision Micro F than for Symfony [16]. As in previous studies, our study showed similar results that PanOptix and FineVision perform better at near distance than Symfony. However, distance and intermediate vision at 80 cm showed comparable visual acuity among the four study groups: the PanOptix group and FineVision group demonstrated UNVA of 0.02 logMAR and 0.05 logMAR, respectively, whereas the Symfony group presented with acuity of 0.21 logMAR at near distance (40 cm). Defocus curves in our study also showed that PanOptix and FineVision had a broad range of depth of focus, whereas Symfony and MiniWell were excellent at defocus power of −0.5 D and −1.0 D. In the direct comparison of PanOptix and FineVision, PanOptix performed better at 60 cm vision.

Regarding MiniWell, numerous studies on in vitro optical quality exist, whereas there are only a few reports directly comparing the clinical outcomes between MiniWell and other multifocal IOLs. Savini et al., compared MiniWell with the bifocal ReSTOR SV25T (Alcon) and concluded that MiniWell performed similarly to ReSTOR SV25T (bifocal IOL) at distance and near vision but was superior at intermediate distances [17]. As in this previous study, our study demonstrated that MiniWell provided good intermediate vision with fair near vision. Defocus curve of MiniWell in our study demonstrated the maintenance of visual performance above 0.1 logMAR at the defocus power of −2.5 D, which is superior to the performance of Symfony.

In reference to refractive outcomes, all four groups in the current study demonstrated satisfactory results. However, the Symfony group presented with myopia, unlike the other groups. This was because the target refraction was set as slightly myopic (from −0.5 D to −0.75 D) for non-dominant eyes. Symfony was reported to provide relatively weak near vision with shorter depth of focus compared to other trifocal lenses in several papers [15,16]. Therefore, we applied mini-monovision to obtain better near vision at the time of surgery. The difference in refractive outcome may have caused a difference between the trend in visual acuity at distant, intermediate, and near in Figure 1 and defocus curve in Figure 2. Visual acuity for distant, intermediate, and near in Figure 1 were measured uncorrected, and defocus curve in Figure 2 was measured after full correction of refraction. 

It is a controversial assertion that Symfony provides better contrast sensitivity results than trifocal IOLs. Theoretically, eyes implanted with Symfony should experience fewer photic phenomena and less loss of contrast sensitivity at distance than those implanted with traditional multifocal diffractive IOLs. According to the manufacturers, the echelette design of Symfony, a proprietary pupil-independent type of diffraction grating, enhances contrast sensitivity using achromatic technology for the correction of chromatic aberration. Meccuci et al., reported that Symfony has better contrast sensitivity than PanOptix and AT LISA tri 839 MP [15]. By contrast, there was no difference in postoperative contrast sensitivity among the four groups in the current study. Previous prospective studies have also failed to demonstrate the superiority of Symfony in contrast sensitivity. Cochener et al., reported that contrast sensitivity was comparable for Symfony, PanOptix, and FineVision [16]. Monaco et al., also demonstrated that Symfony has comparable performance in contrast sensitivity to PanOptix [18]. Our study result regarding contrast sensitivity may have failed to prove existing difference of contrast sensitivity among IOL due to the small number of participants or more myopic refraction of Symfony patients. Further large prospective research with more participants is required to confirm this point.

Total HoA, coma, and trefoil at 4 mm pupil size did not differ among the four study groups. However, SA was different among the study groups. The PanOptix and FineVision groups had more positive SA than the Symfony and MiniWell groups. Investigators believe that this result is attributable to differences in individual IOL specifications; PanOptix and FineVision both have an SA of −0.10 μm, whereas Symfony has an SA of −0.27 μm.

Interestingly, results of the Quality of Vision questionnaire demonstrated that glare, halo, and starburst symptoms were the least in the MiniWell group. This finding may be attributable to the optical quality of MiniWell. Previous studies of in vitro optical quality of MiniWell have shown that it performs better in large pupils [19]. Overall, HoA and SA are not affected by increasing pupil size when MiniWell is used, which is in contrast to the use of Symfony [19]. MiniWell also showed better modulation transfer function performance in larger pupils than Symfony, AT LISA tri 839 MP, and FineVision [20,21]. Hence, in the nighttime, MiniWell would have been able to maintain better visual performance, despite enlarged pupils, than the other IOLs in our study. 

There are some limitations of the present study that need to be considered. First, our study included a relatively small number of patients considering that the performance is affected by various factors such as pupil diameter, corneal aberrations, and visual potential of the patients included. Prediction error would be hard to interpret as the number of eyes are small. We were not able to achieve high statistical power for parameters due to the small number of subjects. Second, HOAs measurements in patients with multifocal IOLs is controversial as these were reported to provide unreliable results in Hartmann-Shack aberrometers [22]. However, OPD-Scan III, which was used in our study, has been reported to have a benefit to measure ocular aberration as Garzón et al., discussed in their recent paper [23]. Additionally, in vitro bench test results of prior studies accord with the results of the current study [19,20,21], so we believe that our postoperative HOAs results are worth reporting. Third, the new generation formulas such as Kane and EVO were not used for the IOL power calculation, as the period during which this study was actually conducted was before the Kane formula or EVO formula became popular. Similarly, the toric versions of PanOptix, Symfony, and MiniWell were not available at the time this study was conducted, which is a limitation of the present study. Fourth, the study was retrospective and is prone to confounding factors such as indication and baseline characteristics. For instance, it is possible that patients with more myopia were offered the lens considered to give the best near vision. In the present study, the FineVision group had longer axial length, and this might have influenced the clinical outcomes and patients reported outcomes. Hyperopic patients could have greater satisfaction on near vision postoperatively and myopic patients could have greater impression for the surgery as they do not need glasses for daily life while have lesser satisfaction for near vision. This is an inherent limitation of this type of study. However, the specifications such as the study setting at a single center, procedures performed by skilled surgeons, consecutive patient enrollment, and uneventful surgeries in all cases, guarantee the uniformity of surgical procedures. In addition, the aforementioned routine examination process for bilateral implantation of multifocal IOLs at the Samsung Medical Center ensures uniform measurements of clinical outcomes in the current study. Moreover, we are the first to compare clinical outcomes of trifocal IOLs and EDOF IOLs in Koreans. Furthermore, there has been no other report regarding clinical outcomes of MiniWell in the Asian population. Therefore, the investigators believe that the current study is clinically significant.

In conclusion, our study presented the characteristics of PanOptix, FineVision, Symfony, and MiniWell, and compared their clinical outcomes. All four IOLs presented satisfactory clinical outcomes. PanOptix and FineVision have excellent near vision performance, whereas Symfony and MiniWell provide excellent intermediate vision. MiniWell induces marginal dysphotopsia compared with the other three IOLs. It could be a good option for those who have large pupils, which makes them prone to dysphotopsia after cataract surgery. For those who require near vision rather than intermediate vision, Symfony should not be used.

## Figures and Tables

**Table 1:** Baseline demographics of the study participants, classified according to IOLs.

Parameters	Trifocal IOLs			EDOF IOLs			*p*-Value *
	PanOptix	FineVision	*p*-Value ^†^	Symfony	MiniWell	*p*-Value ^†^	
Patients (Eyes)	12 (24)	19 (38)		20 (40)	17 (34)		
Age (years)	59.58 ± 6.65	57.58 ± 7.46	0.405	60.35 ± 8.99	62.76 ± 11.79	0.191	0.113
Sex (Male:Female)	4:8	5:14	0.704	10:10	9:8	0.858	0.316
Preoperative							
AXL (mm)	23.96 ± 0.61	24.56 ± 1.75	0.044	24.04 ± 1.31	23.88 ± 1.18	0.096	0.037
ACD (mm)	3.40 ± 0.48	3.32 ± 0.37	0.304	3.29 ± 0.34	3.27 ± 0.38	0.844	0.390
Mean Keratometry (D)	43.73 ± 1.06	44.21 ± 1.31	0.287	43.90 ± 1.45	43.58 ± 1.97	0.745	0.604
Corneal Cylinder (D)	0.76 ± 0.33	1.15 ± 0.49	0.001	0.73 ± 0.30	0.82 ± 0.43	0.421	<0.001
Spherical Equivalent (D)	−0.05 ± 1.60	−2.41 ± 3.67	0.021	−0.96 ± 2.57	−0.02 ± 1.41	0.295	0.034
Refractive Cylinder (D)	−0.91 ± 0.71	−0.89 ± 0.63	0.973	−0.89 ± 0.55	−0.63 ± 0.35	0.099	0.416
Monocular UCVA (logMAR)	0.47 ± 0.45	0.63 ± 0.50	0.147	0.49 ± 0.35	0.33 ± 0.28	0.081	0.017
Monocular BCVA (logMAR)	0.22 ± 0.30	0.24 ± 0.28	0.210	0.35 ± 0.28	0.27 ± 0.17	0.057	0.182
Corneal high order aberration	0.31 ± 0.14	0.27 ± 0.13	0.794	0.35 ± 0.11	0.33 ± 0.10	0.427	0.127
Corneal coma	0.17 ± 0.12	0.16 ± 0.12	0.711	0.20 ± 0.11	0.18 ± 0.10	0.632	0.365
Corneal trefoil	0.13 ± 0.09	0.15 ± 0.11	0.722	0.16 ± 0.10	0.16 ± 0.09	0.530	0.603
Corneal spherical aberration (z12)	0.11 ± 0.06	0.14 ± 0.07	0.156	0.10 ± 0.07	0.13 ± 0.07	0.055	0.121

ACD: anterior chamber depth; AXL: axial length; BCVA: best corrected visual acuity; D: diopter; EDOF: extended depth of focus; IOL: intraocular lens; UCVA: uncorrected visual acuity; BCVA: best-corrected visual acuity; logMAR: logarithm of the minimum angle of resolution. * Overall *p*-value was calculated using Fisher’s exact test for nominal variables and Kruskal–Wallis test for continuous variables. ^†^ *p*-value was calculated using Fisher’s exact test for nominal variables and Mann–Whitney test for continuous variables (PanOptix vs. FineVision, Symfony vs. MiniWell).

**Table 2:** Refractive outcome and predictive error in the four IOL groups.

Parameter	Trifocal IOLs			EDOF IOLs			*p*-Value *
	PanOptix	FineVision	*p*-Value ^†^	Symfony	MiniWell	*p*-Value ^†^	
Spherical Equivalent (D)	−0.02 ± 0.18	0.09 ± 0.34	0.059	−0.26 ± 0.35	−0.04 ± 0.29	0.001	<0.001
Refractive Cylinder (D)	−0.21 ± 0.26	−0.32 ± 0.43	0.611	−0.44 ± 0.39	−0.32 ± 0.25	0.441	0.688
Absolute prediction error							
SRK-T	0.15 (0.07, 0.34)	0.21 (0.14, 0.34)	0.699	0.19 (0.06, 0.43)	0.26 (0.06,0.34)	0.195	0.868
Haigis	0.24 (0.12, 0.35)	0.24 (0.12, 0.41)	0.310	0.25 (0.10, 0.44)	0.15 (0.12, 0.36)	0.822	0.879
Barrett II	0.17 (0.10, 0.23)	0.10 (0.04, 0.42)	0.291	0.21 (0.11, 0.47)	0.17 (0.08, 0.36)	0.634	0.635

D: diopter; EDOF: extended depth of focus; IOL: intraocular lens. Spherical equivalent and refractive cylinder were described as means ± standard deviations, and absolute predictive error was described as median (interquartile range). * Overall *p*-value was calculated using Kruskal–Wallis test. ^†^ *p*-value was calculated using Mann–Whitney test (PanOptix vs. FineVision, Symfony vs. MiniWell). The Symfony group presented with statistically significant results indicating myopia, unlike other groups.

**Table 3:** Ocular higher-order aberrations in the four IOL groups (pupil size = 4.0 mm).

Parameter	Trifocal IOLs			EDOF IOLs			*p*-Value *
	PanOptix	FineVision	*p*-Value ^†^	Symfony	MiniWell	*p*-Value ^†^	
High order aberration	0.233 ± 0.113	0.249 ± 0.117	0.477	0.254 ± 0.087	0.227 ± 0.098	0.162	0.557
Coma	0.061 ± 0.030	0.069 ± 0.044	0.619	0.068 ± 0.032	0.082 ± 0.030	0.205	0.370
Trefoil	0.197 ± 0.118	0.210 ± 0.099	0.566	0.194 ± 0.100	0.1184 ± 0.103	0.780	0.786
Spherical aberration (z12)	0.023 ± 0.018	0.011± 0.029	0.117	−0.023 ± 0.035	−0.027 ± 0.026	0.755	<0.001

IOL: intraocular lens. * Overall *p*-value was calculated using Kruskal–Wallis test. ^†^ *p*-value was calculated using Mann–Whitney test (PanOptix vs. FineVision, Symfony vs. MiniWell).

## Data Availability

The datasets used and/or analyzed during the current study are available from the corresponding author upon reasonable request.

## Supplementary Materials

The following supporting information can be downloaded at: https://www.mdpi.com/article/10.3390/jcm11195729/s1, Table S1: The mean and standard deviation of corrected binocular visual acuity measured for defocus curve at postoperative 3 months, Table S2: The mean and standard deviation of contrast sensitivity under photopic condition, mesopic condition without glare, and mesopic condition with glare at postoperative 3 months, Table S3: The power analyses for analysis of variance performed.

## References

[1] Agresta B., Knorz M.C., Kohnen T., Donatti C., Jackson D. (2012). Distance and near visual acuity improvement after implantation of multifocal intraocular lenses in cataract patients with presbyopia: A systematic review. J. Refract. Surg..

[2] Mesci C., Erbil H., Ozdoker L., Karakurt Y., Bilge A.D. (2010). Visual acuity and contrast sensitivity function after accommodative and multifocal intraocular lens implantation. Eur. J. Ophthalmol..

[3] Calladine D., Evans J.R., Shah S., Leyland M. (2012). Multifocal versus monofocal intraocular lenses after cataract extraction. Cochrane Database Syst. Rev..

[4] Cochener B., Lafuma A., Khoshnood B., Courouve L., Berdeaux G. (2011). Comparison of outcomes with multifocal intraocular lenses: A meta-analysis. Clin. Ophthalmol..

[5] Hutz W.W., Eckhardt H.B., Rohrig B., Grolmus R. (2008). Intermediate vision and reading speed with array, Tecnis, and ReSTOR intraocular lenses. J. Refract. Surg..

[6] Jonker S.M., Bauer N.J., Makhotkina N.Y., Berendschot T.T., van den Biggelaar F.J., Nuijts R.M. (2015). Comparison of a trifocal intraocular lens with a +3.0 D bifocal IOL: Results of a prospective randomized clinical trial. J. Cataract Refract. Surg..

[7] Cochener B. (2016). Prospective Clinical Comparison of Patient Outcomes Following Implantation of Trifocal or Bifocal Intraocular Lenses. J. Refract. Surg..

[8] Pedrotti E., Bruni E., Bonacci E., Badalamenti R., Mastropasqua R., Marchini G. (2016). Comparative Analysis of the Clinical Outcomes With a Monofocal and an Extended Range of Vision Intraocular Lens. J. Refract. Surg..

[9] Sudhir R.R., Dey A., Bhattacharrya S., Bahulayan A. (2019). AcrySof IQ PanOptix Intraocular Lens Versus Extended Depth of Focus Intraocular Lens and Trifocal Intraocular Lens: A Clinical Overview. Asia Pac. J. Ophthalmol..

[10] Hwang S., Lim D.H., Hyun J., Kim M.J., Chung T.Y. (2018). Myopic Shift after Implantation of a Novel Diffractive Trifocal Intraocular Lens in Korean Eyes. Korean J. Ophthalmol..

[11] Yang C.M., Lim D.H., Hwang S., Hyun J., Chung T.Y. (2018). Prospective study of bilateral mix-and-match implantation of diffractive multifocal intraocular lenses in Koreans. BMC Ophthalmol..

[12] Noh H., Yoo Y.S., Shin K.Y., Lim D.H., Chung T.Y. (2021). Comparison of penetrating femtosecond laser-assisted astigmatic keratotomy and toric intraocular lens implantation for correction of astigmatism in cataract surgery. Sci. Rep..

[13] Schmitz S., Dick H.B., Krummenauer F., Schwenn O., Krist R. (2000). Contrast sensitivity and glare disability by halogen light after monofocal and multifocal lens implantation. Br. J. Ophthalmol..

[14] Paik D.W., Park J.S., Yang C.M., Lim D.H., Chung T.Y. (2020). Comparing the visual outcome, visual quality, and satisfaction among three types of multi-focal intraocular lenses. Sci. Rep..

[15] Mencucci R., Favuzza E., Caporossi O., Savastano A., Rizzo S. (2018). Comparative analysis of visual outcomes, reading skills, contrast sensitivity, and patient satisfaction with two models of trifocal diffractive intraocular lenses and an extended range of vision intraocular lens. Graefes Arch. Clin. Exp. Ophthalmol..

[16] Cochener B., Boutillier G., Lamard M., Auberger-Zagnoli C. (2018). A Comparative Evaluation of a New Generation of Diffractive Trifocal and Extended Depth of Focus Intraocular Lenses. J. Refract. Surg..

[17] Savini G., Schiano-Lomoriello D., Balducci N., Barboni P. (2018). Visual Performance of a New Extended Depth-of-Focus Intraocular Lens Compared to a Distance-Dominant Diffractive Multifocal Intraocular Lens. J. Refract. Surg..

[18] Monaco G., Gari M., Di Censo F., Poscia A., Ruggi G., Scialdone A. (2017). Visual performance after bilateral implantation of 2 new presbyopia-correcting intraocular lenses: Trifocal versus extended range of vision. J. Cataract Refract. Surg..

[19] Camps V.J., Tolosa A., Pinero D.P., de Fez D., Caballero M.T., Miret J.J. (2017). In Vitro Aberrometric Assessment of a Multifocal Intraocular Lens and Two Extended Depth of Focus IOLs. J. Ophthalmol..

[20] Dominguez-Vicent A., Esteve-Taboada J.J., Del Aguila-Carrasco A.J., Ferrer-Blasco T., Montes-Mico R. (2016). In vitro optical quality comparison between the Mini WELL Ready progressive multifocal and the TECNIS Symfony. Graefes Arch. Clin. Exp. Ophthalmol..

[21] Dominguez-Vicent A., Esteve-Taboada J.J., Del Aguila-Carrasco A.J., Monsalvez-Romin D., Montes-Mico R. (2016). In vitro optical quality comparison of 2 trifocal intraocular lenses and 1 progressive multifocal intraocular lens. J. Cataract Refract. Surg..

[22] Charman W.N., Montés-Micó R., Radhakrishnan H. (2008). Problems in the measurement of wavefront aberration for eyes implanted with diffractive bifocal and multifocal intraocular lenses. J. Refract. Surg..

[23] Garzón N., García-Montero M., López-Artero E., Poyales F., Albarrán-Diego C. (2019). Influence of trifocal intraocular lenses on standard autorefraction and aberrometer-based autorefraction. J. Cataract Refract. Surg..

