# Supplementary material for: Clinical Outcomes after Bilateral Implantation of Trifocal Diffractive Intraocular Lenses and Extended Depth of Focus Intraocular Lenses
Source: J Clin Med. 2022 Sep 27;11(19):5729. doi: 10.3390/jcm11195729 (PMC9571716; doi:10.3390/jcm11195729)
Supplement: Supplementary file 1 [file jcm-11-05729-s001.zip › jcm-1895871-supplementary/Table S1_4IOLs.pdf]

Table S1. The mean and standard deviation of corrected binocular visual acuity measured for defocus curve at postoperative 3 months.

**Diopter**	**+2.0**	**+1.5**	**+1.0**	**+0.5**
**PanOptix**	0.42 ± 0.11	0.27 ± 0.15	0.12 ± 0.13	-0.02 ± 0.13
**FineVision**	0.15 ± 0.15	0.07 ± 0.11	-0.02 ± 0.09	-0.07 ± 0.11
**Symfony**	0.31 ± 0.23	0.17 ± 0.20	0.06 ± 0.15	-0.05 ± 0.08
**MiniWell**	0.13 ± 0.19	0.05 ± 0.16	0.00 ± 0.15	-0.05 ± 0.07
**Diopter**	**0.0**	**-0.5**	**-1.0**	**-1.5**
**PanOptix**	-0.11 ± 0.09	-0.02 ± 0.09	0.00 ± 0.13	-0.06 ± 0.12
**FineVision**	-0.09 ± 0.10	-0.05 ± 0.10	0.00 ± 0.11	-0.01 ± 0.11
**Symfony**	-0.10 ± 0.07	-0.11 ± 0.09	-0.06 ± 0.12	-0.01 ± 0.13
**MiniWell**	-0.09 ± 0.07	-0.09 ± 0.06	-0.06 ± 0.07	-0.07 ± 0.07
**Diopter**	**-2.0**	**-2.5**	**-3.0**	**-3.5**
**PanOptix**	-0.04 ± 0.10	0.02 ± 0.09	0.14 ± 0.13	0.24 ± 0.16
**FineVision**	-0.05 ± 0.11	0.00 ± 0.12	0.09 ± 0.11	0.24 ± 0.14
**Symfony**	0.02 ± 0.12	0.11 ± 0.11	0.25 ± 0.14	0.38 ± 0.15
**MiniWell**	-0.02 ± 0.08	0.05 ± 0.10	0.12 ± 0.11	0.26 ± 0.14
