# Supplementary material for: Clinical Outcomes after Bilateral Implantation of Trifocal Diffractive Intraocular Lenses and Extended Depth of Focus Intraocular Lenses
Source: J Clin Med. 2022 Sep 27;11(19):5729. doi: 10.3390/jcm11195729 (PMC9571716; doi:10.3390/jcm11195729)
Supplement: Supplementary file 1 [file jcm-11-05729-s001.zip › jcm-1895871-supplementary/Table S2_4IOLs.pdf]

Table S2. The mean and standard deviation of contrast sensitivity under photopic condition, mesopic condition without glare, and mesopic condition with glare at postoperative 3 months.

**Photopic condition**	**3cpd**	**6cpd**	**12cpd**	**18cpd**
**PanOptix**	1.75 ± 0.06	1.95 ± 0.14	1.57 ± 0.15	1.03 ± 0.21
**FineVision**	1.79 ± 0.08	1.97 ± 0.21	1.67 ± 0.23	1.14 ± 0.18
**Symfony**	1.69 ± 0.16	1.91 ± 0.16	1.65 ± 0.18	1.10 ± 0.19
**MiniWell**	1.76 ± 0.10	1.96 ± 0.19	1.73 ± 0.25	1.12 ± 0.20
**Mesopic condition without glare**	**3cpd**	**6cpd**	**12cpd**	**18cpd**
**PanOptix**	1.49 ± 0.18	1.55 ± 0.24	1.08 ± 0.36	0.64 ± 0.32
**FineVision**	1.60 ± 0.18	1.49 ± 0.18	1.07 ± 0.33	0.54 ± 0.25
**Symfony**	1.49 ± 0.20	1.41 ± 0.37	0.93 ± 0.29	0.44 ± 0.28
**MiniWell**	1.51 ± 0.14	1.60 ± 0.21	1.20 ± 0.56	0.70 ± 0.44
**Mesopic condition with glare**	**3cpd**	**6cpd**	**12cpd**	**18cpd**
**PanOptix**	1.69 ± 0.08	1.89 ± 0.20	1.43 ± 0.24	1.00 ± 0.21
**FineVision**	1.67 ± 0.14	1.78 ± 0.15	1.49 ± 0.17	0.95 ± 0.27
**Symfony**	1.60 ± 0.20	1.76 ± 0.19	1.43 ± 0.31	0.93 ± 0.27
**MiniWell**	1.73 ± 0.15	1.88 ± 0.22	1.56 ± 0.31	1.04 ± 0.26

cpd=cycle per degree
