# Supplementary material for: Clinical Outcomes after Bilateral Implantation of Trifocal Diffractive Intraocular Lenses and Extended Depth of Focus Intraocular Lenses
Source: J Clin Med. 2022 Sep 27;11(19):5729. doi: 10.3390/jcm11195729 (PMC9571716; doi:10.3390/jcm11195729)
Supplement: Supplementary file 1 [file jcm-11-05729-s001.zip › jcm-1895871-supplementary/Table S3_4IOLs.pdf]

Table S3. The power analyses for analysis of variance performed.

**Parameters analyzed**	**Effect size**	**Power**
**Visual outcome**		
– Best corrected visual acuity	0.205	0.221
– Uncorrected distant visual acuity	0.204	0.218
– Uncorrected intermediate (80cm) visual acuity	0.139	0.121
– Uncorrected intermediate (60cm) visual acuity	0.398	0.703
– Uncorrected near (40cm) visual acuity	0.581	0.967
**Refractive Outcome**		
– Postoperative spherical equivalent	0.551	0.992
– Postoperative refractive cylinder	0.161	0.199
– Absolute prediction error by SRK-T	0.171	0.208
– Absolute prediction error by Haigis	0.164	0.194
– Absolute prediction error by Barrett II	0.251	0.419
**Contrast sensitivity**		
– Photopic, 3 cycle per degree	0.350	0.499
– Photopic, 6 cycle per degree	0.137	0.107
– Photopic, 12 cycle per degree	0.260	0.289
– Photopic, 18 cycle per degree	0.201	0.184
– Mesopic without glare, 3 cycle per degree	0.271	0.256
– Mesopic without glare, 6 cycle per degree	0.251	0.224
– Mesopic without glare, 12 cycle per degree	0.266	0.249
– Mesopic without glare, 18 cycle per degree	0.303	0.315
– Mesopic with glare, 3 cycle per degree	0.276	0.323
– Mesopic with glare, 6 cycle per degree	0.297	0.371
– Mesopic with glare, 12 cycle per degree	0.189	0.167
– Mesopic with glare, 18 cycle per degree	0.150	0.120
**High order aberration**		
– High order aberration	0.103	0.092
– Coma	0.200	0.232
– Trefoil	0.096	0.086
– Spherical aberration (z12)	0.610	0.989
**Quality of vision**		
– Glare	0.516	0.871
– Halo	0.557	0.921
– Starburst	0.408	0.664
– Hazy vision	0.146	0.119
– Blurring	0.217	0.218
**Spectacle Independence**		

– Near	0.503	0.882
– Intermediate	0.243	0.286
– Far	0.115	0.095
**Satisfaction**		
– Near	0.217	0.225
– Intermediate	0.124	0.100
– Far	0.347	0.528
